# Class III antiarrhythmic drugs amiodarone and dronedarone impair K_IR_2.1 backward trafficking

**DOI:** 10.1111/jcmm.13172

**Published:** 2017-04-19

**Authors:** Yuan Ji, Hiroki Takanari, Muge Qile, Lukas Nalos, Marien J.C. Houtman, Fee L. Romunde, Raimond Heukers, Paul M.P. van Bergen en Henegouwen, Marc A. Vos, Marcel A.G. van der Heyden

**Affiliations:** ^1^ Division of Heart & Lungs Department of Medical Physiology UMCU Utrecht The Netherlands; ^2^ Department of Physiology Faculty of Medicine in Pilsen Charles University in Prague Pilsen Czech Republic; ^3^ Cell Biology Department of Biology Science Faculty Utrecht University Utrecht The Netherlands

**Keywords:** inward rectifier, K_IR__2.1_, degradation, lysosome, amiodarone, dronedarone

## Abstract

Drug‐induced ion channel trafficking disturbance can cause cardiac arrhythmias. The subcellular level at which drugs interfere in trafficking pathways is largely unknown. K_IR_2.1 inward rectifier channels, largely responsible for the cardiac inward rectifier current (I_K__1_), are degraded in lysosomes. Amiodarone and dronedarone are class III antiarrhythmics. Chronic use of amiodarone, and to a lesser extent dronedarone, causes serious adverse effects to several organs and tissue types, including the heart. Both drugs have been described to interfere in the late‐endosome/lysosome system. Here we defined the potential interference in K_IR_2.1 backward trafficking by amiodarone and dronedarone. Both drugs inhibited I_K__1_ in isolated rabbit ventricular cardiomyocytes at supraclinical doses only. In HK‐KWGF cells, both drugs dose‐ and time‐dependently increased K_IR_2.1 expression (2.0 ± 0.2‐fold with amiodarone: 10 μM, 24 hrs; 2.3 ± 0.3‐fold with dronedarone: 5 μM, 24 hrs) and late‐endosomal/lysosomal K_IR_2.1 accumulation. Increased K_IR_2.1 expression level was also observed in the presence of Na_v_1.5 co‐expression. Augmented K_IR_2.1 protein levels and intracellular accumulation were also observed in COS‐7, END‐2, MES‐1 and EPI‐7 cells. Both drugs had no effect on K_v_11.1 ion channel protein expression levels. Finally, amiodarone (73.3 ± 10.3% *P* < 0.05 at −120 mV, 5 μM) enhanced I_KIR__2.1_ upon 24‐hrs treatment, whereas dronedarone tended to increase I_KIR__2.1_ and it did not reach significance (43.8 ± 5.5%, *P* = 0.26 at −120 mV; 2 μM). We conclude that chronic amiodarone, and potentially also dronedarone, treatment can result in enhanced I_K__1_ by inhibiting K_IR_2.1 degradation.

## Introduction

Proper ion channel expression and function is one of the cornerstones of normal heart function. Unequal ion distribution between the intra‐and extracellular compartment in concert with ion specific voltage‐sensitive channels in the plasma membrane determines action potential formation. The stable and negative resting membrane potential in between action potentials results from the activity of the inward rectifying ion channels of the *KCNJ* gene family 1. In the heart, the K_IR_2.1 channel protein, encoded by *KCNJ2*, is the main contributor to ventricular I_K1_. K_IR_2.1 loss of function has been associated with Andersen–Tawil syndrome, characterized by action potential prolongation, and thus QT‐lengthening on the ECG. Furthermore, patients experience periodic paralysis and mild episodes of cardiac arrhythmia 2. In contrast, gain‐of‐function mutations are associated with QT shortening and atrial fibrillation 3. Besides its important function in the heart, K_IR_2.1 proteins also contribute to inward rectifier currents in skeletal and smooth muscle, and several neuronal cell types 4. In Andersen–Tawil syndrome patients, association with the occurrence of increased U‐waves on the ECG has been found 5. Pharmacological inhibition of KCNJ channels by barium has also been associated with more apparent U‐waves 6. In a study on the presence and amplitudes of U‐waves associated with loss‐ and gain‐of‐function mutations in KCNJ2 patients at normokalemic conditions, the authors speculate that at least a part of the U‐wave is inversely correlated with the amount of I_K1_ 7.

K_IR_2.1 ion channel trafficking is a strictly regulated process that can be divided into forward (anterograde; towards the plasma membrane) and backward (retrograde; from the plasma membrane) trafficking events 8. K_IR_2.1 channels become internalized *via* a clathrin‐mediated pathway and subsequently travel towards the lysosome, where the channels ultimately become degraded *via* an initial discrete cleavage step that removes the N‐terminus 9, 10. Interference in lysosomal degradation and upstream trafficking events by specific inhibitors results in increased K_IR_2.1 expression levels, and most likely by saturation of the endocytotic machinery, also in increased I_K1_ densities 9, 10. Also clinical drugs can have significant effects on ion channel trafficking and this can lead to severe adverse effects 8. Among the variety of affected channel proteins, the K_IR_2.1 channel internalization and degradation is sensitive for disturbances by, although old, clinical drugs like chlorpromazine and chloroquine 9, 10, 11.

Amiodarone is a class III antiarrhythmic, based on the benzofuran structure used in atrial and ventricular fibrillation therapy 12. Amiodarone is a multichannel blocker affecting delayed rectifier I_Kr_, sodium channel and L‐type calcium currents. Amiodarone therapy is known for its many adverse effects on the ocular, neurological, dermatological, thyroid, gastrointestinal, pulmonary, cardiac and liver systems 13, 14, 15. Some studies demonstrate detrimental effects of amiodarone on cargo trafficking through the late‐endosome/lysosome compartments, which could partly explain the plethora of side effects 16, 17, 18. Amiodarone has been shown to inhibit the degradation of lung surfactant protein A *in vitro* and *in vivo* 16. Dronedarone is a synthetic analogue of amiodarone developed to preserve antiarrhythmic properties with less adverse effects, especially thyroid and pulmonary toxicity 19. Compared with amiodarone, dronedarone is less lipophilic and has a much shorter half‐life (1–2 *versus* 30–55 days). Nevertheless, also dronedarone appears to interfere in normal late‐endosome/lysosome function 17. Chronic amiodarone therapy has been associated with the appearance of prominent U‐waves 20, 21, 22, which may allude to a potential disturbance of I_K1_. Currently, it is unknown whether amiodarone and dronedarone interfere in the process of K_IR_2.1 trafficking, in particular its degradation, which was therefore investigated in the current study.

## Materials and methods

### Rabbit ventricular cardiomyocyte isolation

Animal care and experimental procedures were in accordance with the ‘European Directive for the Protection of Vertebrate animals used for Experimental and Scientific Purpose, European Community Directive 2010/63/EU’ and were approved by the Committee for Experiments on Animals of the Utrecht University, the Netherlands.

Ventricular rabbit cardiomyocytes were isolated by enzymatic digestion using a Langendorff set‐up identical to that described previously 23.

### Cell culture

HEK293 cells expressing C‐terminal GFP‐tagged murine K_IR_2.1 (HK‐KWGF cells) were cultured as described before 9, 24. Mouse P19 embryonal carcinoma‐derived germ layer cell lines END‐2, MES‐1 and EPI‐7 cells 25, 26, COS‐7, HEK293t, HEK‐hERG 27 and Ex‐293 28 cells were cultured in DMEM (Lonza, Breda, the Netherlands) supplemented with 10% FCS (Sigma‐Aldrich, Zwijndrecht, the Netherlands), 2 mM L‐glutamine (Lonza), and 50 U/ml penicillin and 50 mg/ml streptomycin (both Lonza). In time course experiments, cells were seeded and harvested on identical days.

In COS‐7 western blot experiments, cells were transfected using linear polyethylenimine (PEI). In short, PEI (Mw 25,000 Polysciences Inc., Eppelheim, Germany) was dissolved in water at 0.323 g/l. PEI solution was subsequently adjusted to pH 8.0, sterilized using filtration and freeze‐thawed four times. Aliquots of PEI stock solution were stored at −20°C. For each transfection, 2.5 μg plasmid DNA was added to a 150 mM NaCl solution, total volume 150 μl. 20 μl of PEI stock solution was also added to a 150 mM NaCl solution, total volume 150 μl. Both solutions were mixed, incubated at room temperature for 20 min. and subsequently added to the cells. Medium was replaced at 16 hrs post‐transfection. In immunofluorescence microscopy experiments, HEK293t, END‐2, MES‐1 and EPI‐7 cells were transfected with human K_IR_2.1 + Rab7‐GFP or K_IR_2.1 alone using Lipofectamine (Invitrogen, Breda, the Netherlands) according to the manufacturer's protocol.

### Drugs

Amiodarone (cat. no. 8357 lot AR20569) and dronedarone (cat. no. SR33589B lot 7963) (both Sanofi Recherche, Montpellier, France) were dissolved in DMSO at 50 mM.

### Immunohistochemistry and confocal microscopy

HK‐KWGF cells were cultured on Ø 15‐mm cover slips, pre‐coated with poly‐L‐lysine (Sigma‐Aldrich). END‐2, MES‐1, EPI‐7 and HEK293t cells were cultured on Ø 15‐mm cover slips, pre‐coated with 0.1% gelatin. Cells were rinsed with PBS supplemented with 1 mM Ca^2+^ and 1 mM Mg^2+^ and fixed with 3% paraformaldehyde, pH 7.4. Permeabilization was performed with 0.5% Triton X‐100 in PBS and 50 mM PBS–glycine was used as quenching agent. To block non‐specific interaction sites, NET‐gel was applied on the cells. Then cells were incubated overnight with the primary antibodies K_IR_2.1 (for END‐2, MES‐1, EPI‐7 and HEK293t cells (1:250; Santa Cruz Biotechnology, Heidelberg, Germany, cat. no. sc‐18708), LAMP‐1 (1:200; BD Bioscience Pharmingen, Breda, The Netherlands) or EEA1 (1:1000; BD Bioscience Pharmingen) (both for HK‐KWGF cells) in NET‐gel. Cell nuclei were stained with 40,6‐diamidino‐2‐phenylindole (DAPI) (1:50.000; Molecular Probes, Leiden, The Netherlands) during secondary antibody incubation. A five times 5 min. wash step procedure was done with NET‐gel before and after incubation with donkey antimouse DyLight secondary antibody (1:250; Jackson ImmunoResearch Laboratories Inc., West Baltimore Pike West Grove, PA, USA) or donkey anti‐goat Alexa Red (1:400; Jackson ImmunoResearch Laboratories Inc.). The cover slips were mounted with Vectashield (Vector Laboratories Inc. Burlingame, CA, USA), and confocal images were obtained using a Zeiss Axiovert 200 M confocal microscope (Carl Zeiss Microscopy GmbH, Germany) equipped with a 63× water immersion objective (NA 1.2) plus 2× digital zoom. Excitation was performed with an air‐cooled Argon ion laser (LASOS, RMC 7812Z, 488 nm) for GFP and a HeNE (LASOS, SAN 7450A, 543 nm) laser for DyLight. Colocalization between K_IR_2.1‐GFP, EEA1, and LAMP‐1, and K_IR_2.1 and Rab7‐GFP, was quantified by determining the Pearson coefficient (*r*) with the Costes automated threshold method provided by the JACoB plugin for the ImageJ software 29.

### Western blotting

Following treatment, cells were harvested in lysis buffer (20 mM HEPES, pH 7.6, 125 mM NaCl, 10% (v/v) glycerol, 1 mM EDTA, 1 mM EGTA, 1 mM dithiothreitol, 1% (v/v) Triton X‐100). Subsequently, 20 μg protein lysate was separated by 7% or 10% SDS‐PAGE and blotted onto nitrocellulose membrane. Blots were blocked with 5% (w/v) non‐fat milk powder for detection with GFP antibody (1:500; Santa Cruz Biotechnology, cat. no. sc9996) or K_v_11.1 antibody (1:3000; Alomone Labs, Jerusalem, Israel, cat. no. APC062) or 5% egg yolk (v/v) for K_IR_2.1 antibody (1:250; Santa Cruz Biotechnology, cat. no. sc‐18708) in TBST (20 mM Tris–Cl, pH 8.0, 150 mM NaCl, 0.05% (v/v) Tween‐20) for 1 hr at room temperature. Donkey antimouse or anti‐goat (Jackson ImmunoResearch, cat. nos. 715‐065‐137 and 705‐035‐003, respectively) horseradish peroxidase secondary antibody was subsequently used. Standard ECL Prime procedure was used for final detection (GE Healthcare Life Sciences, Eindhoven, the Netherlands).

### Electrophysiology

In ventricular rabbit cells, I_K1_ was measured by patch clamp experiments in whole‐cell mode using an Axon amplifier controlled by pClamp9.2 software (Molecular Devices, Sunnyvale, CA, USA). Experiments were performed at 37°C using temperature control (Cell MicroControls, Norfolk, VA, USA). Cardiomyocytes were put in the chamber and superfused with normal Tyrode's solution (mM) (140 NaCl, 5 KCl, 6 HEPES, 6 glucose, 1.8 CaCl_2_, 1 MgCl_2_, pH 7.4 with NaOH). Borosilicate glass pipettes were made with a Sutter P‐2000 puller (Sutter Instrument, Novato, CA, USA) and had a pipette resistant of 2–3 MΩ when filled with pipette solution (mM) (110 KCl, 10 EGTA, 10 HEPES, 4 K_2_‐ATP, 5.17 CaCl_2_, 1.42 MgCl_2_, pH 7.2 with KOH). The voltage protocol for I_K1_ measurements was as follows: holding potential was set to −80 mV, and a prepulse at −40 mV for 200 ms was applied to inactivate native sodium current. I_K1_ was elicited by 1‐s step pulses from −120 mV to 30 mV by 10 mV step increments.

HK‐KWGF cells were grown on 0.1% gelatin (Bio‐Rad, Veenendaal, the Netherlands) coated Ø 12‐mm cover slips. I_KIR2.1_ from single cells was recorded in whole‐cell voltage clamp mode using an Axopatch 200B amplifier and a Digidata 1322A digitizer and recorded with pCLAMP 9.2 software. Signals were low‐pass‐filtered at 2 kHz and sampled at 4 kHz. Measurements were taken at 37°C in a temperature‐controlled perfusion chamber filled with tyrode solution containing (in mM) NaCl 140, KCl 5.4, CaCl_2_ 1.8, MgCl_2_ 1, glucose 6, HEPES 6, pH 7.4/NaOH. Pipettes were pulled on a Sutter Instrument P‐2000 laser micropipette puller and had a resistance of 1.5–3 MΩ when filled with pipette solution, containing (in mM) K‐gluconate 125, KCl 10, EGTA 5, CaCl_2_ 0.6, MgCl_2_ 2, HEPES 5, Na_2_ATP 4, pH 7.2/KOH. HK‐KWGF cells were kept at a holding potential of ‐40 mV and 1‐s test pulses were applied ranging from −120 mV to +30 mV with increments of 10 mV.

Steady‐state currents from both cell types were analysed using Clampfit 9.2 software (Molecular Devices) and corrected for membrane capacitance to determine current density.

### Statistics

Data were analysed using GraphPad Prism version 6.00 for Windows (GraphPad Software, La Jolla, California USA) or Origin 8 (Microcal Software, Northampton, MA, USA) for rabbit cardiomyocyte measurements. For normally distributed data, Student's *t*‐test or anova for paired samples with Tukey's HSD post hoc or Bonferroni correction for multiple comparisons was used, while nonparametric data were analysed using Wilcoxon rank‐sum test and Friedman's test with Dunn's multiple comparison test. Results are presented as mean ± S.E.M. Values of *P* < 0.05 were considered significant.

## Results

**Figure 1:**
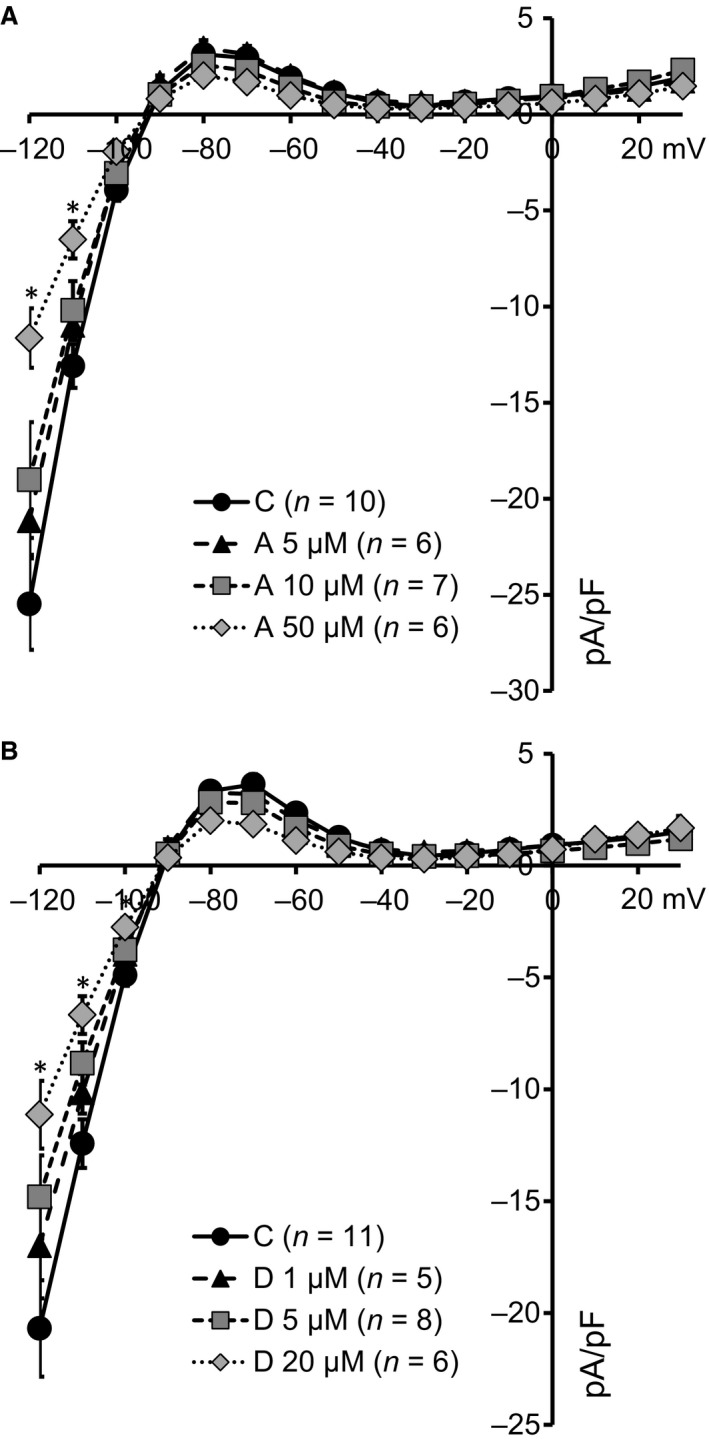
Acute application of supraclinical concentrations of amiodarone and dronedarone inhibits I_k1_ in rabbit left ventricular cardiomyocytes. (**A**) I_K1_ current–voltage relationships of cardiomyocytes superfused with 5 μM (triangles, *N* = 6), 10 μM (squares, *N* = 7) and 50 μM (diamonds, *N* = 6) amiodarone (A) display dose‐dependent decreases in I_K1_ reaching significance for 50 μM (at −120 and −110 mV) only. C depicts time‐matched controls (*N* = 10). (**B**) I_K1_ current–voltage relationships of cardiomyocytes superfused with 1 μM (triangles, *N* = 5), 5 μM (squares, *N* = 8) and 20 μM (diamonds, *N* = 6) dronedarone (D) display dose‐dependent decreases in I_K1_ reaching significance for 20 μM (at −120, −110 and −100 mV) only. C depicts time‐matched controls (*N* = 11). *P < 0.05.

Amiodarone and dronedarone are known to have I_K1_ blocking capacities in guinea pig ventricular cardiomyocytes 30, 31, albeit that their respective IC_50_ values of >20 μM and >30 μM are beyond maximal plasma levels obtained from patients (approximately 5 μM for amiodarone and 0.3 μM for dronedarone) 19, 32. Using rabbit ventricular cardiomyocytes, we were able to confirm these results as depicted in Figure 1A and B. Block at −120 mV was 17.0 ± 1.4%, 25.4 ± 4.0% and 54.3 ± 7.2% for 5, 10 and 50 μM amiodarone, respectively. Outward current block at −80 mV was 17.5 ± 2.3% and 35.7 ± 6.0% for 10 and 50 μM amiodarone, respectively. Similar levels of inhibition were observed with dronedarone (block at −120 mV of 17.6 ± 2.5%, 28.4 ± 3.6% and 46.2 ± 6.3%; block at −80 mV of 2.3 ± 0.3%, 15.1 ± 2.8% and 40.1 ± 7.0% for 1, 5 and 20 μM, respectively).

**Figure 2:**
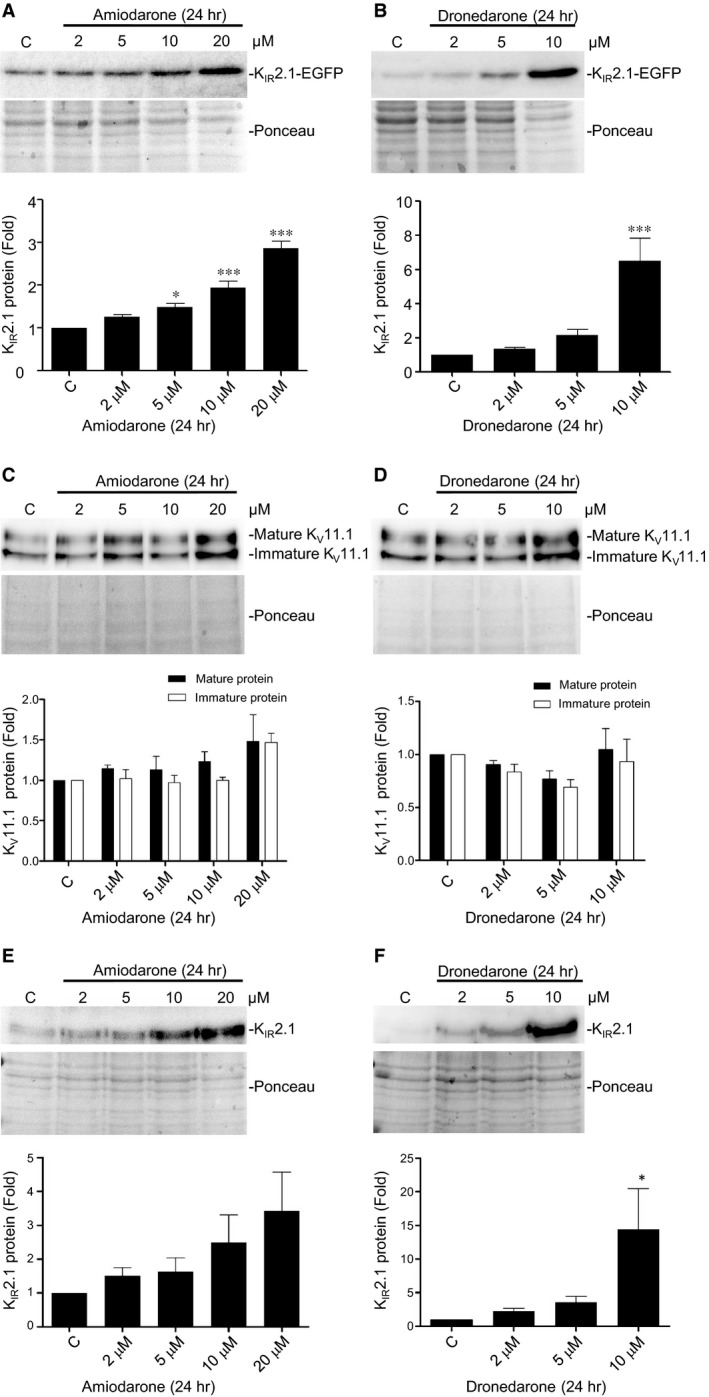
Amiodarone and dronedarone induce dose‐dependent increases in K_IR_2.1‐GFP expression, independent of Na_v_1.5 expression whereas K_v_11.1 expression levels are not affected. (**A** and **B**) Western blot analysis of K_IR_2.1‐GFP expression in HK‐KWGF cells treated with 2, 5, 10 or 20 μM amiodarone or 2, 5 or 10 μM dronedarone for 24 hrs. C indicates control (untreated) cells. Ponceau staining is used as loading control. Averaged data from eight (amiodarone) and seven (dronedarone) independent experiments, respectively, are depicted in bar graphs in the lower part of both panels.(**C** and **D**) Western blot analysis of K_v_11.1 expression in HEK‐hERG cells treated with 2, 5, 10 or 20 μM amiodarone or 2, 5 or 10 μM dronedarone for 24 hrs. C indicates control (untreated) cells. Ponceau staining is used as loading control. Averaged data from 3 independent experiments are depicted in bar graphs in the lower part of both panels. (**E** and **F**) Western blot analysis of K_IR_2.1 expression in Ex293 cells treated with 2, 5, 10 or 20 μM amiodarone or 2, 5 or 10 μM dronedarone for 24 hrs. C indicates control (untreated) cells. Ponceau staining is used as loading control. Averaged data from three (amiodarone) and five (dronedarone) independent experiments are depicted in bar graphs in the lower part of both panels. *P < 0.05; ***P < 0.001.

We next assessed effects of chronic treatment with amiodarone and dronedarone on K_IR_2.1 expression in our previously described model system for K_IR_2.1 channel trafficking, HK‐KWGF cells 9, 10, 11. Both amiodarone and dronedarone resulted in dose‐dependent increase in total K_IR_2.1 expression as established by Western blotting (Fig. 2A and B). In these assays, the strongest effects were reached with 20 μM amiodarone (2.9 ± 0.2‐fold) and 10 μM dronedarone (6.1 ± 1.5), respectively. No effects on mRNA levels were found by quantitative PCR (1.00 ± 0.02 *versus* 0.92 ± 0.02 and 1.02 ± 0.01 for control, 10 μM amiodarone and 5 μM dronedarone, respectively). In contrast, amiodarone and dronedarone were unable to increase mature and immature K_v_11.1 expression in stably transfected HEK293 cells (Fig. 2C and D).

Finally, we tested whether increased K_IR_2.1 expression levels are dependent upon coexpression of Na_v_1.5 expression, a cardiac ion channel that has previously been shown to associate with K_IR_2.1 and which combined expression demonstrates reciprocal modulation 33. Ex‐293 cells, a HEK293 cell line both expressing K_IR_2.1 and Na_v_1.5 28, displayed a dose‐dependent increase in K_IR_2.1 expression upon treatment with either amiodarone or dronedarone (Fig. 2E and F). Strongest effects were observed with 20 μM amiodarone (3.4 ± 1.2‐fold) and 10 μM dronedarone (14.44 ± 6.0).

**Figure 3:**
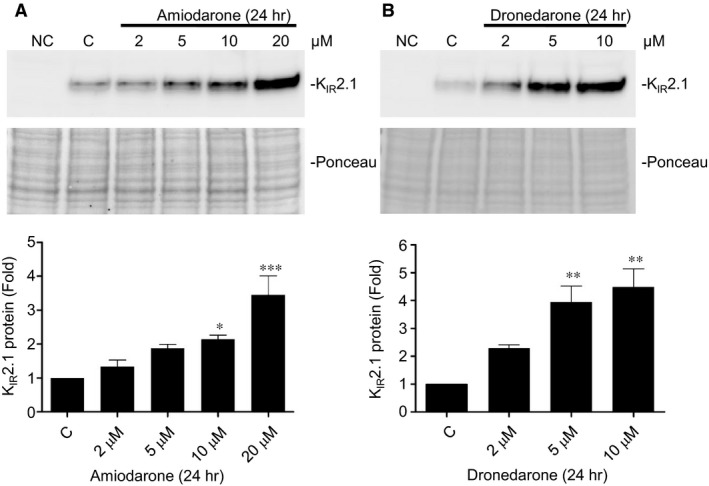
Amiodarone and dronedarone induce dose‐dependent increases in K_IR_2.1 expression in COS‐7 cells. (**A** and **B**) Western blot analysis of K_IR_2.1 expression in COS‐7 cells treated with 2, 5, 10 or 20 μM amiodarone or 2, 5 or 10 μM dronedarone for 24 hrs. C indicates control (untreated) cells. Ponceau staining is used as loading control. Averaged data from three independent experiments are depicted in bar graphs in the lower part of both panels. *P < 0.05; **P < 0.01; ***P < 0.001.

To determine whether the increased K_IR_2.1‐GFP expression levels is cell line specific or depends on the GFP tag, experiments were repeated in transiently transfected COS‐7 cells. Under these conditions, similar effects were seen for amiodarone and dronedarone on the non‐tagged human K_IR_2.1 (Fig. 3A and B). Strongest effects in COS‐7 cells were observed with 20 μM amiodarone (3.4 ± 0.6‐fold) and 10 μM dronedarone (4.5 ± 0.7).

**Figure 4:**
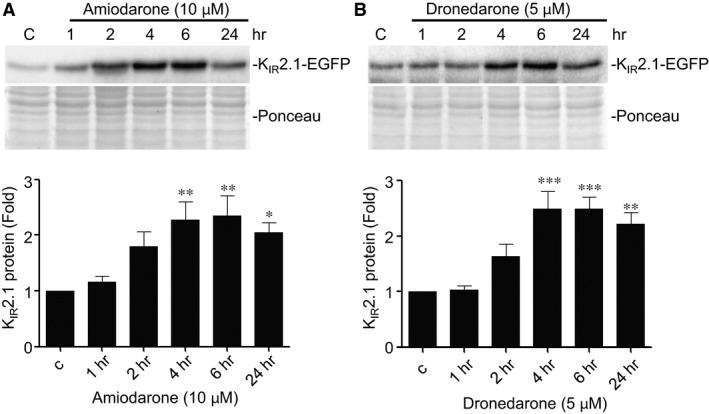
Amiodarone and dronedarone induce time‐dependent increases in K_IR_2.1‐GFP expression. Western blot analysis of K_IR_2.1‐GFP expression in HK‐KWGF cells treated for 1, 2, 4, 6 and 24 hrs with 10 μM amiodarone or 5 μM dronedarone. C indicates control (untreated) cells. Ponceau staining is used as loading control. Averaged data from eight (amiodarone) and ten (dronedarone) independent experiments, respectively, are shown in bar graphs in the lower part of both panels. *P < 0.05; **P < 0.01; ***P < 0.001.

Significant enhanced expression of K_IR_2.1 in HK‐KWGF cells was seen from 4 hrs following drug application. Maximal response rates were observed after 4–6 hrs (Fig. 4A and B).

**Figure 5:**
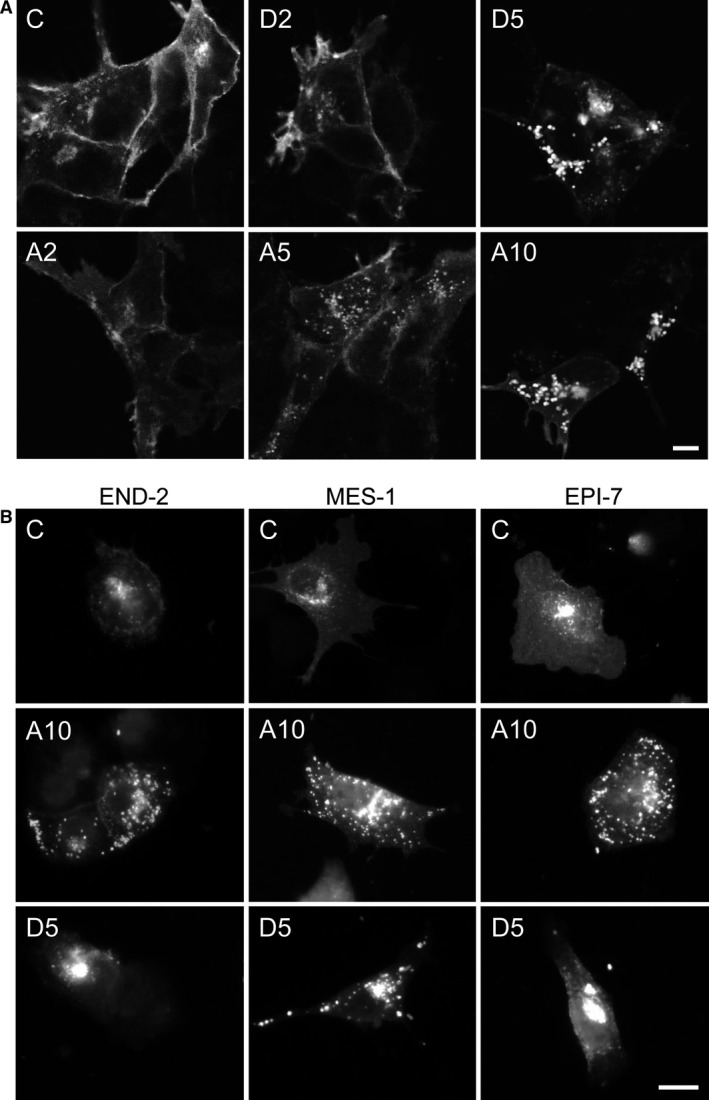
Amiodarone and dronedarone induce dose‐dependent intracellular K_IR_2.1 accumulation. (**A**) K_IR_2.1‐GFP localization in control (C) (untreated) and HK‐KWGF cells treated for 24 hrs with 2 (D2) or 5 (D5) μM dronedarone, or 2 (A2), 5 (A5) or 10 (A10) μM amiodarone. (**B**) K_IR_2.1 localization in control (C) (untreated) and END‐2, MES‐1 and EPI‐7 cells treated for 24 hrs with 10 (A10) μM amiodarone or 5 μM (D5) dronedarone**.** Scale bars represent 5 μm.

Immunofluorescence microscopy revealed dose‐dependent accumulation of K_IR_2.1‐GFP (Fig. 5A) in a pattern resembling that of bafilomycin A1 and chloroquine treatment 10. No intracellular accumulation was seen with 2 μM amiodarone or dronedarone, while relatively small aggregates were seen with 5 μM amiodarone and large aggregates were observed with 10 μM amiodarone or 5 μM dronedarone (Fig. 5A). In order to exclude that K_IR_2.1‐GFP accumulation in response to amiodarone and dronedarone is cell type specific or depends on the GFP tag, we made use of mouse P19 embryonal carcinoma‐derived END‐2, MES‐1 and EPI‐7 cells representing the three different germ layers 34 that were transiently transfected with non‐tagged human K_IR_2.1. Amiodarone at 10 μM induced clear intracellular aggregates similar as observed in HK‐KWGF cells (Fig. 5B). Furthermore, dronedarone at 5 μM induced intracellular K_IR_2.1 accumulation in MES‐1 cells. In END‐2 and EPI‐7 cells, dronedarone appeared to induce larger aggregates (Fig. 5B).

**Figure 6:**
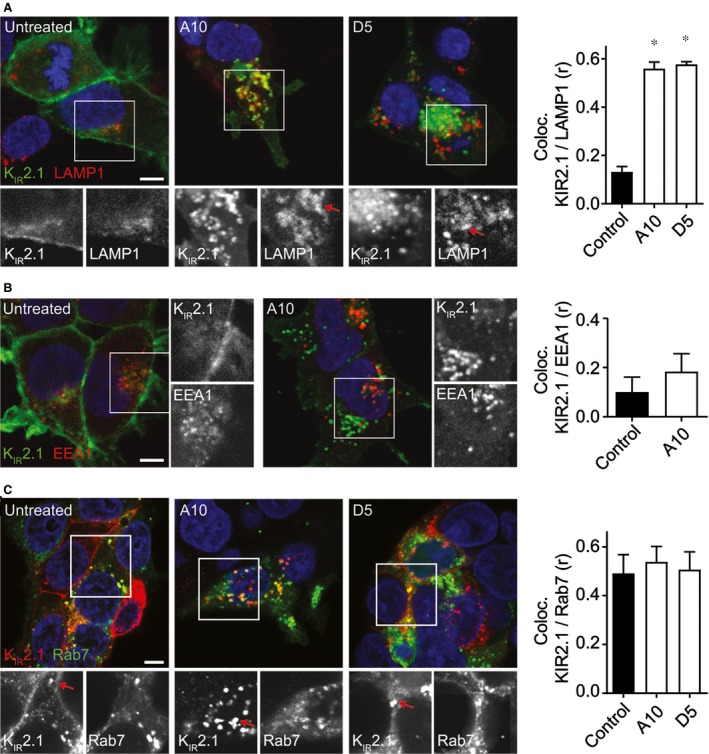
(**A**) Costaining of K_IR_2.1‐GFP and LAMP1 in control (untreated) and cells treated with 10 μM amiodarone (A10) or 5 μM dronedarone (D5). Merged pictures are presented in colour. Individual staining patterns of the boxed parts are given in the lower six panels in b/w. Red arrows indicate regions of colocalization. Pearson coefficient of colocalization is presented as bars on the right. (**B**) Costaining of K_IR_2.1‐GFP (green) and EEA1 (red) in control (untreated) and cells treated with 10 μM amiodarone (A10). Individual staining patterns of the boxed parts are given in the right panels in b/w. Pearson coefficient of colocalization is presented as bars on the right. (**C**) Costaining of K_IR_2.1 (red) and Rab7‐GFP (green) in control (untreated) and cells treated with 10 μM amiodarone (A10) or 5 μM dronedarone (D5). Individual staining patterns of the boxed parts are given in the lower six panels in b/w. Pearson coefficient of colocalization is presented as bars on the right. Scale bars represent 5 μm. *P < 0.05.

An increase in K_IR_2.1‐GFP costaining for lysosomes (LAMP1) was observed following 10 μM amiodarone or 5 μM dronedarone (Pearson coefficient 0.13 ± 0.02, 0.56 ± 0.03 (*P* < 0.05) and 0.58 ± 0.01 (*P* < 0.05) for control, 10 μM amiodarone and 5 μM dronedarone, respectively) (Fig. 6A). Costaining for early endosomes (EEA1) revealed no increase in colocalization following 10 μM amiodarone (0.10 ± 0.06 *versus* 0.18 ± 0.07 (n.s.) for control and 10 μM amiodarone) (Fig. 6B). In cells cotransfected with non‐tagged K_IR_2.1 and Rab7‐GFP (late endosome), no change in colocalization was observed in response to 10 μM amiodarone or 5 μM dronedarone (Pearson coefficient 0.49 ± 0.08, 0.54 ± 0.06 (n.s.) and 0.51 ± 0.07 (n.s.) for control, 10 μM amiodarone and 5 μM dronedarone, respectively) (Fig. 6C).

**Figure 7:**
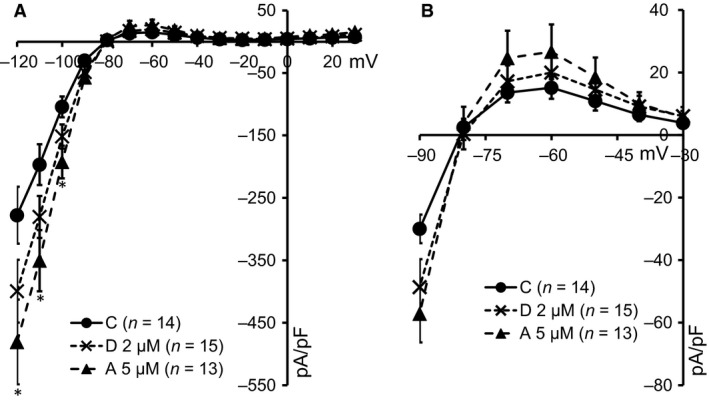
Twenty four‐hours treatment with amiodarone and dronedarone increases functional K_IR_2.l expression. (**A**) Current–voltage relationship of I_KIR2.1_ in control cells (C) (filled circles, *N* = 14) and cells treated with either 2 μM dronedarone (D) (crosses, *N* = 15) or 5 μM amiodarone (A) (triangles, *N* = 13). *Amiodarone effects reach significance (*P* < 0.05) at −120, −110 and −100 mV. (**B**) Enlargement of panel A from membrane voltage between −90 and −30 mV indicating a trend in outward current increase upon amiodarone and dronedarone treatment.

We suggested that the intracellular accumulation of K_IR_2.1‐GFP protein could result in saturation of upstream trafficking pathways which may result in enhanced current levels, as seen before with the lysosomal inhibitor chloroquine 9 and the clathrin‐mediated internalization inhibitor dynasore 10. Cells were treated for 24 hrs with either 2 μM dronedarone or 5 μM amiodarone, and I_K1_ densities were compared to their non‐treated counterparts (Fig. 7A and B). Chronic dronedarone treatment resulted in a slight trend towards increased I_KIR2.1_ densities for the inward (43.8 ± 5.5%, *P* = 0.26 at −120 mV) and a non‐significant increase in outward (32.0 ± 7.8%, *P* = 0.83 at −60 mV) current components. 24‐hrs treatment with amiodarone resulted in a significant increase in the inward current component at −120, −110 and −100 mV of 73.3 ± 10.3%, 78.0 ± 10.9% and 84.4 ± 11.5%, respectively, whereas a non‐significant increased outward current (75.9 ± 24.9%, *P* = 0.38 at −60 mV) was observed.

## Discussion

Amiodarone is known for its hepatic and pulmonary adverse effects in patients. This is associated with the occurrence of lysosomal structural abnormalities such as lamellar lysosomal inclusion bodies 35, 36. Less is known on the effects of amiodarone on muscle cell lysosome morphology and function. Several case reports demonstrate the occurrence of skeletal muscle vacuolarization with or without the presence of inclusion bodies upon chronic amiodarone therapy, interpreted as lysosomal defects by the authors 37, 38. In myocardial fibres from the left and right ventricle, and right atrium derived from dogs chronically treated with amiodarone, abnormal lysosomal structures with often dense lamellar inclusion bodies were found 39. Similar ‘autophagic vacuoles’ were observed in isolated rat ventricular myocytes chronically treated with amiodarone *in vitro* 40, 41. Morissette *et al*. demonstrated that amiodarone application resulted in vacuolar sequestration and evolved towards persistent macroautophagy in macrophages, smooth muscle cells and HEK293 cells 42. Dronedarone shows strong similarities to amiodarone with respect to induction of the formation of cellular vacuoles containing lamellar bodies (lysosomal structures) as demonstrated in alveolar macrophages 43.

We found that amiodarone and dronedarone treatment increased K_IR_2.1 expression and intracellular accumulation, most likely in late endosomes and lysosomes, in several different cell lines. Interestingly, compared with chloroquine treatment that results in lysosomal accumulation of full‐length and a discrete N‐terminally cleaved K_IR_2.1 protein, only accumulation of the full‐length product is seen with amiodarone and dronedarone. Therefore, either the majority of the K_IR_2.1 accumulates in pre‐lysosomal compartments, which is in line with the findings of Picolli *et al*. 17 who describe that amiodarone and dronedarone do not affect early endosome function, but interferes in the late compartments of the endocytotic pathway, or these compounds interfere in protease function responsible for the N‐terminal K_IR_2.1 cleavage. The latter explanation is in line with findings of Buratta *et al*. 44 who describe that specific cathepsins display altered processing in some cell types upon amiodarone treatment. Whatever the exact mechanism, our findings for K_IR_2.1 are in line with those of Baritussio *et al*. 16, who demonstrated that amiodarone inhibits surfactant protein A degradation that normally takes place in the lysosomal compartment.

As amiodarone treatment correlates with the induction of autophagocytosis, especially upon longer treatment (>24 hrs), we cannot exclude the possibility that a part of the intracellular K_IR_2.1 accumulation occurs in non‐functioning, due to the amiodarone and dronedarone acid buffering capacity, macroautophagosomes 42. This may contribute to the observed colocalization of K_IR_2.1 with LAMP‐1. Finally, expression level of the K_v_11.1 potassium channel protein is not increased by amiodarone or dronedarone, once more demonstrating channel specificity in trafficking pathways and their (patho)physiological regulation 45.

When considering potassium ion channel trafficking with respect to the action of amiodarone and dronedarone, only few data are available in the literature. Taniguchi *et al*. 46 found no effect of amiodarone on I_Ks_ channel trafficking in Chinese hamster ovary cells. In the hERG‐Lite assay 47, amiodarone inhibits hERG surface expression which may result from impaired forward or enhanced backward trafficking or translation interference. We and others showed that backward trafficking of hERG and K_IR_2.1 channels follows different pathways, which makes them react differently to a number of drugs 11. We showed that amiodarone and dronedarone also affect K_IR_2.1 trafficking differently than that for hERG channels. In cardiomyocytes isolated from guinea pigs treated with amiodarone for 7 days, decreased I_K1_, I_Ks_ and I_Kr_ densities were found 48. In contrast, in cardiomyocytes from mice treated with amiodarone for 6 weeks, no differences in I_K1_ densities, in neither *KCNJ2* nor *KCNJ12* transcript levels, were observed 49. For now, it is unclear to what extent and by what mechanisms amiodarone and dronedarone affect potassium ion channel trafficking *in vivo* which warrants future research.

## Conflict of interest

The authors confirm that there are no conflict of interests.
